# Parallel Selection Revealed by Population Sequencing in Chicken

**DOI:** 10.1093/gbe/evv222

**Published:** 2015-11-13

**Authors:** Saber Qanbari, Michael Seidel, Tim-Mathias Strom, Klaus F.X. Mayer, Ruedi Preisinger, Henner Simianer

**Affiliations:** ^1^Animal Breeding and Genetics Group, Georg-August University, Göttingen, Germany; ^2^Institute of Plant Genome and Systems Biology, Helmholtz Zentrum München, Neuherberg, Germany; ^3^Institute of Human Genetics, Helmholtz Zentrum München, Munich, Germany; ^4^Lohmann Tierzucht GmbH, Cuxhaven, Germany

**Keywords:** improvement, selective sweep, domestication, population differentiation

## Abstract

Human-driven selection during domestication and subsequent breed formation has likely left detectable signatures within the genome of modern chicken. The elucidation of these signatures of selection is of interest from the perspective of evolutionary biology, and for identifying genes relevant to domestication and improvement that ultimately may help to further genetically improve this economically important animal. We used whole genome sequence data from 50 hens of commercial white (WL) and brown (BL) egg-laying chicken along with pool sequences of three meat-type chicken to perform a systematic screening of past selection in modern chicken. Evidence of positive selection was investigated in two steps. First, we explored evidence of parallel fixation in regions with overlapping elevated allele frequencies in replicated populations of layers and broilers, suggestive of selection during domestication or preimprovement ages. We confirmed parallel fixation in *BCDO2* and *TSHR* genes and found four candidates including *AGTR2*, a gene heavily involved in “Ascites” in commercial birds. Next, we explored differentiated loci between layers and broilers suggestive of selection during improvement in chicken. This analysis revealed evidence of parallel differentiation in genes relevant to appearance and production traits exemplified with the candidate gene *OPG*, implicated in Osteoporosis, a disorder related to overconsumption of calcium in egg-laying hens. Our results illustrate the potential for population genetic techniques to identify genomic regions relevant to the phenotypes of importance to breeders.

## Introduction

Chicken is the most intensively farmed animal on earth and is a major food source with billions of birds used in meat and egg production each year. Many of the features of the chicken genome and its biology make it an ideal organism for studies in development and evolution, along with applications in agriculture and medicine (Burt 2005).

It is postulated that chickens (*Gallus domesticus*) were primarily domesticated from a wild form called red jungle fowl (*Gallus gallus*) (Fumihito et al. 1996; Hillel et al. 2003), a bird that still runs wild in most of Southeast Asia, although it still is in debate whether the origin of chicken is monophyletic or polyphyletic (Nishibori et al. 2005; Ericsson et al. 2008). During domestication and subsequent breed formation, greatly influenced by human activities, chickens have adapted in morphology, physiology, and behavior to increase yield, fertility, and other processes (Ericsson et al. 2014). This has likely left detectable signatures within the genome of modern chicken and can be used to screen the genome for genes involved in recent adaptation.

Our understanding of the chicken genome has been mostly transformed through two landmark events in recent years. First, assembling the whole genome sequence as a reference for the chicken genome (Hillier et al. 2004) and second, characterizing 2.8 million unique single nucleotide polymorphisms (SNPs) in the genome of domesticated chicken (Wong et al. 2004), suggestive of a higher nucleotide diversity compared with humans. This SNP panel later formed the basic platform to create SNP genotyping chips for the use in whole genome-based selection studies (Johansson et al. 2010; Elferink et al. 2012; Wragg et al. 2012), motivated by the desire to localize genes implicated in recent adaptation. A 600K SNP array was recently developed (Kranis et al. 2013) that provides an increased genotyping resolution and higher throughput. There were also efforts to study selection in chicken by implementing pooled sequencing strategies (Rubin et al. 2010; Qanbari et al. 2011). Although SNP arrays suffer notably from ascertainment bias and low marker resolution, pooled sequencing guarantees a high resolution, but produces uncertain allelic frequency profiles and remains blind to the individual genotypes and local haplotype structures. Many selection signals may, therefore, have remained undetected so far.

In this study, we investigate evidence of recent selection for traits relevant to domestication and subsequent improvement in replicated populations of egg-laying and meat-type chicken. Using whole genome sequence information in replicated populations shaped by a parallel selection regime provides additional power to detect selection missed by previous studies. We employ multiple statistics to scan the genome and find evidence of positive selection during pre- and postbreeding ages in chicken exemplified by several candidate genes colocalized with major quantitative trait loci (QTLs).

## Materials and Methods

### Genetic Material, Sequencing, and Variant Calling

For the purpose of this study, we sequenced 50 female birds of two commercial white (WL, *n* = 25) and brown (BL, *n* = 25) egg-laying populations of the LOHMANN Tierzucht GmbH at 10 × coverage. The sequenced birds are pure parent lines of white and brown egg commercial hybrids that respectively lay up to 290 and 270 eggs in 68 weeks of age (http://www.ltz.de/). The paired-end reads with a read length of 101 bp were mapped against the current reference genome assembly Galgal4 using the Burrows-Wheeler aligner (Version 0.6.1) with setting of default parameters. Duplicate reads were masked during postprocessing using the Picard tool set (version 1.84, http://broadinstitute.github.io/picard/, last accessed November 28, 2015). SNPs were called simultaneously in all 50 individuals’ alignments using default parameters in FreeBayes (verison v9.9.2-22-gc283d6d; http://arxiv.org/abs/1207.3907). The resulting vcf files were sorted and indexed to allow rapid random access.

To ensure the highest possible data quality, a series of filters were employed to remove lower quality SNPs and insecure genotypes for individuals. We kept polymorphisms with a minimum Phred score of 20 (99% accuracy) as an acceptable error rate. To preclude overrepresentation of repetitive sequences, we only used polymorphisms in range 2 σ read depth. The final sequence panel involved more than 9,300,000 SNPs with an average intermarker space of 107 bp in both populations.

### Broiler Sequence Data

We used a data set by Rubin et al. (2010) available at the European Nucleotide Archive website (http://www.ebi.ac.uk/ena/) under the study accession number SRP001870. This data set is composed of 35 bp reads obtained by SOLiD sequencing technology of genomic DNA pools of unrelated chicken. We remapped the reads from three broiler populations named as CB1, CB2, and “High” against the chicken reference genome Galgal4. A set of SNPs with positions allocated to the previous reference genome assembly galGal3 was available from an associated project. Those SNPs were excised in their galGal3 context (2× 50-bp flanking) as artificial reads and saved with each of both possible bases at the SNP position. The full set of sequence pairs was then mapped to the new reference sequence gelGal4 using vmatch with parameters chosen so only complete matches (all 101 bp) were reported. In a final filtering step, only alignments where one of the two reads had a unique hit were reported. In total, ∼3% of SNPs were lost during remapping including those that did not map uniquely or had less than 50 bp of flanking sequence available.

### Detecting Positive Selection

Evidence of positive selection was investigated in two steps and through multiple statistics. First, we explored evidence of parallel fixation in regions with overlapping elevated allele frequencies (AF ≥ 0.8) in replicated populations of layers and a pool of three broilers assuming that recent selection was responsible for parallel fixation at similar genes, suggestive of selection during domestication or preimprovement ages. Next, we explored differentiation of loci between two pools of layers and broilers using Fst metric (Reynolds et al. 1983) as a representation of parallel diverging selection for target traits during improvement in chicken. We further employed the integrated Haplotype Homozygosity Score (iHS) to examine the local structure of haplotype (Voight et al. 2006) and investigated Heterozygosity (Het), supposed to be reduced in regions affected by selection. To reduce locus-to-locus variation in the inference of selection, we averaged single SNP values for overlapping windows of 40 kb stepping in 5 kb across the genome.

It is shown that recombination rate could affect local extent of differentiation (Keinan and Reich 2010). We examined this in chicken as microchromosomes show substantially higher recombination than macrochromosomes. We found a subtle difference between micro- (mean Fst = 0.186) and macrochromosomes (mean Fst = 0.184), and given that the study tries to find outlier signals of differentiation, we followed the identical windowing approach across the genome.

## Results and Discussion

### Exploring Footprints of Domestication

Domestication process has driven the genetic change in parallel among current breeds/populations, and therefore can be studied through replicated populations. This scenario assumes that same alleles are responsible for domestication relevant traits in each replicate and true signals generated by selection would overlap across the populations. Such a strategy benefits from the fact that genetic drift alone did not drive allele frequency changes. This concept has been successfully applied in several recent studies to explore evidence for parallel evolution either using genotyping arrays (Elferink et al. 2012; Wragg et al. 2012) or resequencing pools in chicken (Rubin et al. 2010) and other domestic species (Rubin et al. 2012; Carneiro et al. 2014), but not in a resolution provided by population sequencing.

**Fig. 1.—:**
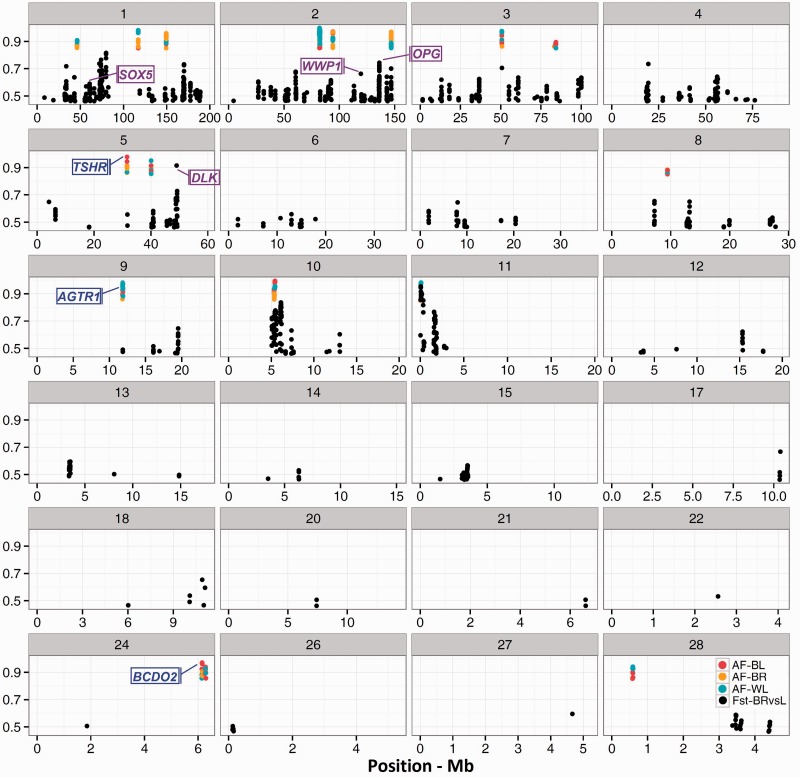
Visualization of parallel selection candidates along GGA1-28. Brown (BL), Orange (BR), and cyan (WL) dots display regions with elevated allele frequency (AF > 0.85), measured in parallel in two layers and a pool of three broiler populations. The black dots depict the genome-wide map of differentiation between layers and broilers represented by Fst metric. Both metrics are plotted in overlapping windows of 40 kb in steps of 5 kb. Genes in blue and purple represent candidates revealed respectively by analyses of parallel fixation and divergence.

To examine this hypothesis, we scanned the genome for regions with elevated allele frequencies separately in WL and BL and explored its overlap with a pool of three broiler populations. To facilitate comparison of genomic regions across populations, we averaged the allele frequencies in windows of 40 kb overlapped in steps of 5 kb. Evidence of the parallel fixation was then assumed for chromosomal fragments with AF > 0.85 in all populations. As predicted by selective sweep theory, although the selected allele increases in prevalence, the hitchhiking effect drags adjacent alleles to the higher frequencies. Accordingly, the windows exceeding the threshold often appeared in contiguous tracts as extended chromosomal regions and ultimately clustered in six peaks of cofixation (fig. 1 and table 1).

**Table 1:** Candidate Gene/Regions Detected as Signal of Parallel Fixation

Chromosome	Position (bp)	AF_WL_	AF_BL_	AF_BR_	Gene	Function
1	46162081–46327076	0.90	0.87	0.86	Gene desert	
1	117000428–117030951	0.98	0.86	0.91	Gene desert	
1	149367651–149494049	0.95	0.88	0.95	*LOC101748868*	Uncharacterized
2	81956167–82594173	0.99	0.89	0.90	*VSTM2A*	A predictedtarget-SNARE gene
2	94091060–94204500	0.92	0.97	0.96	*CCDC102B*	
2	146767959–146879733	0.93	0.91	0.97	*TSNARE1*	Neurobehavioral
3	50672559–50816899	0.97	0.95	0.91	*C7ORF10*	
3	84015975–84072368	0.89	0.87	0.86	Gene desert	
5	31562907–31623166	0.90	0.98	0.91	*GJD2*	Expressed in brain and retina
5	40044067–40104591	0.95	0.91	0.89	*TSHR*	Reproduction
8	9411058–9463004	0.86	0.88	0.87	Gene desert	
9	11798419–11901393	0.98	0.98	0.86	*AGTR1*	Ascites, hypertension, and susceptibility to fatty liver disease
10	5316513–5444763	0.95	0.99	0.90	*APBA2*	Neurobehavioral
11	35837–98851	0.98	0.98	0.85	*LTB4R*	
24	6113460–6173359	0.88	0.97	0.90	*BCDO2*	Yellow skin color
28	579151–594628	0.94	0.90	0.86	*SPPL2B*	

First, we sought the allele frequency profile in the region of the *BCDO2* and *TSHR* loci, two well-documented examples of positive selection in the domestic chicken, as proof of principle demonstrating that this approach could localize gene regions that underwent parallel fixation. The *TSHR* and *BCDO2* genes are shown to control respectively the reproductive machinery (Rubin et al. 2010) and yellow skin color (Ericsson et al. 2008) in modern chicken, and current populations of broiler and laying birds are supposed to have undergone fixation for certain alleles in these loci. We observed strong signals of parallel fixation over *BCDO2* (AF_WL_ = 0.88, AF_BL_ = 0.97, AF_BR_ = 0.90) and *TSHR* (AF_WL_ = 0.95 and AF_BL_ = 0.91, AF_BR_ = 0.89) (fig. 1), and the regions perfectly overlapped the previously defined selective sweeps. This provides the evidence that both genes have been fixed during domestication, before the improvement in commercial chicken started.

One striking observation of this analysis was parallel fixation overlapping the angiotensin II type 1 receptor (*AGTR1*) gene (AF_WL_ = 0.98, AF_BL_ = 0.98, AF_BR_ = 0.86). In humans, *AGTR1* is a strong candidate for the pulmonary arterial hypertension (PAH, Chung et al. 2009). Ascites, the industry term for PAH in chickens, is a result of heavy diets that stimulate fast growth rate and causes significant mortality in broiler chickens (Pavlidis et al. 2007). Variants of the *AGTR1* gene are shown to be associated with the Ascites in chicken (Dey 2012; Wideman et al. 2013). Ascites, however, is a disease of modern days in the poultry industry and therefore we speculate that fixation in *AGTR1* has occurred very recently, after chickens were maintained and fed in captivated systems. Another signal overlaps VSTM2A (AFWL = 0.99, AFBL = 0.89, AFBR = 0.90), a predicted target-SNARE gene that was already reported by Rubin et al. (2010). We also noticed a strong signal standing by the *GJD2* gene (AF_WL_ = 0.90, AF_BL_ = 0.98, AF_BR_ = 0.91) that plays a role in retinal neurotransmission, and is shown to be a major candidate for the vision refractive errors and myopia (Hornbeak and Young, 2009). However, no evidence supports the evolution of vision perception in birds during domestication. Further research on the *AGTR1* and *GJD2* genes would be required to address potential adaptation process in chicken domestication. This comparison further revealed parallel fixation in two genomic regions including a gene desert and an uncharacterized protein (table 1).

### Exploring Footprints of Improvement

Genomic regions with a high degree of genetic differentiation between populations are also indicative of selection. The formation of paralleled commercial breeds during an extremely short time period is likely a result of rapid fixation of alleles/haplotypes under intensive artificial selection. Under this scenario, in replicated commercial populations that have been under similar breeding regime inter- and intrapopulations (e.g., egg layers vs. broilers), an overlapping cluster of differentiated alleles may reflect parallel divergence of beneficial allele. Therefore, statistics based on Fst can serve as efficient tools to identify footprints of parallel divergence. Several recent studies have revealed evidence for parallel divergence at the same loci in replicated population comparisons (Kijas et al. 2012; Qanbari et al. 2012; Petersen et al. 2013; Xu et al. 2015; among others). We performed a genome-wide differentiation scan to localize variants probably affecting egg versus meat production traits. To this purpose, the average allelic frequencies derived from pooling of three broiler populations were compared against average allelic frequency in two laying populations, assuming that loci with extreme differentiation reflect signals of parallel divergence.

The empirical genome-wide distribution of Fst indicates that recent selection has severely operated on the genome of commercial birds (genome-wide Fst = 0.18, standard deviation = 0.08), when being compared with the sequence-based estimates of 0.05–0.07 in human continental populations (1000 Genomes Project Consortium 2010) or <0.01 among African populations (Bhatia et al. 2011). Single site values of Fst were accumulated in overlapping windows of 40 kb (in steps of 5 kb) and resulted in a total of 139,005 windows across the genome. Evidence of positive selection was then assumed for windows in the extreme top 1% (Fst > 0.46) of the empirical distribution. Most significant windows were clustered together as extended chromosomal fragments resulting in 170 differentiated regions (supplementary table S1, Supplementary Material online). These results based on sequencing entire genome provide a detailed map of differentiated loci, some colocalized with previously suggested QTLs.

### Genes Putatively Under Parallel Divergence

In domestic poultry, a strong negative relationship is observed between body weight and reproductive effectiveness (Muir and Aggrey 2003). Laying hens have been bred for maximum egg production rather than meat yield and due to the negative correlation, are supposed to be under inevitable selection for lower body weight and vice versa. Therefore, genes of interest in this analysis are growth rate and muscularity suggestive of positive selection in rapidly growing meat-type birds versus traits relevant to egg production in layers. For clarity and based on a priori interest, we divided genes into three groups in line with breeding goals together with appearance traits and discuss each group under separate heading. However, as most genes have pleiotropic effects, selection may possibly act on other functional effects of the genes than those highlighted here. In the following sections, we highlight some results from these analyses.

#### Selection Candidates for Laying Traits

**Fig. 2.—:**
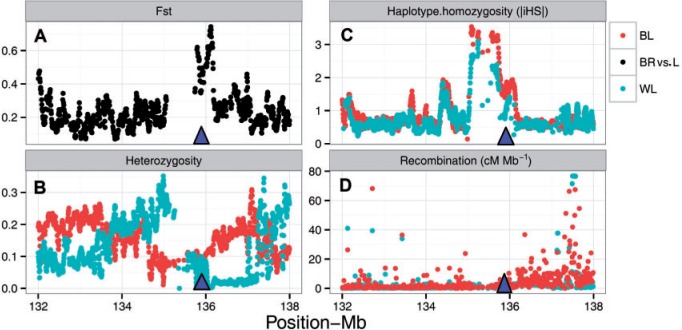
A graphical representation of candidate *OPG* gene region on GGA2. Local parallel divergence between broilers (BR) and layers (L) are depicted as Fst in black. Sliding-window analyses of Fst, |iHS|, and heterozygosity across the 4-Mb interval are plotted as overlapping 40 kb windows in steps of 5 kb. Recombination rates are plotted in 10 kb windows and triangle shows the position of gene.

Today’s commercial laying hens have been selectively bred to produce more eggs per hen housed per year. For instance, commercial hybrids of WL and BL populations in this study produce respectively up to 318 and 312 eggs per year of laying (for more details see http://www.ltz.de/). This unnaturally high level of productivity is metabolically taxing, often causing hens to suffer from production diseases. Bone is the metabolic reservoir for calcium used in egg shell production, and moving calcium from bone to egg shell leaves the hen prone to osteoporosis, subsequent bone fragility, and bone fractures. Among the top selection candidates, we noticed four genes associated with bone biology and disorders. For instance, strong evidence of a candidate selective sweep reflected by 41 contiguous windows and extending over 1 Mb on GGA2 was observed in the region harboring the 2 exostosin (*EXT1* and *EXT1L*) and the Osteoprotegerin (*TNFRSF11B*) genes (max Fst = 0.74, *P* ≤ 0.00071) (figs. 1 and 2). Exostosins are implicated in a variety of bone disorders (Wuyts and Van Hul 2000) and Osteoprotegerin is a key negative regulator of osteoclastogenesis, secreted by osteoblasts cells. In humans, polymorphisms within the *OPG* gene have been widely studied and associated with bone mineral density, osteoporosis, and fracture risk (Richards et al. 2008; Mencej-Bedrač et al. 2011; among others). Osteoporosis is a progressive loss in structural bone and is a common problem in caged egg-laying strains of hens (Whitehead and Fleming 2000). Welfare issues associated with osteoporosis have become more urgent due to the increasing use of battery cages. In addition to animal welfare concerns, osteoporosis causes major economic loss in the egg-laying industry (Schreiweis et al. 2005).

Another differentiation signal in this group overlaps *CLDNS11* gene on GGA9 (max Fst = 0.64, *P* = 0.00132). Claudins regulate paracellular transport of ions, solutes, and water and are the primary proteins responsible for the formation of tight junctional strands in osteoblasts. Recent evidence suggests a significant role for the Claudins, in the regulation of bone mineral density (Thorleifsson et al. 2009).

#### Selection Candidates for Growth Rate and Muscularity

Meat-type birds have been intensively selected for growth rate and body composition, which has reduced the age at market weight. For instance, most commercial broilers could weigh up to 2.77 kg at 47 days of age with feed efficiency rate dropped to 1.89 of live weight (http://www.nationalchickencouncil.org/). A particularly interesting differentiation peak in this group occurs in the region harboring the *WWP1* (max Fst = 0.50, *P* = 0.00525) gene. An R441Q missense mutation in this gene is shown to be responsible for the chicken muscular dystrophy (Matsumoto et al. 2008). Increasing evidence demonstrates that selection has markedly altered the muscle functioning in rapidly growing meat birds. In contrast, selection for high rates of egg laying has not affected muscle (Sandercock et al. 2009). An elevated differentiation occurred between broiler and layer birds of different populations, suggesting that adverse variants or haplotypes of *WWP1* have probably been under parallel selection.

Among the differentiated regions, we also noticed an extensive chromosomal tract spanning over 24 contiguous windows on GGA5 that harbors the Delta-like protein 1 (*DLK1*) gene (max Fst = 0.91, *P* = 0.00004). *DLK1* is implicated in the muscle hypertrophy observed in mice and callipyge sheep (White et al. 2008). Polar overdominant inheritance of a *DLK1* polymorphism is also associated with growth and fatness in pigs (Kim et al. 2004). It is also shown that *DLK1* has a significantly greater expression in muscles of broilers compared with layers (Shin et al. 2009). It could be argued that intensive selection regime has fixed the favored variants of this gene in parallel across broiler populations. Another signal in this group embeds the Forkhead box protein O1 (*FOXO1*, max Fst = 0.730966, *P* = 0.00077) gene, which plays role in myogenic growth and differentiation. Transgenic mice and rats overexpressing *FOXO1* weigh less than the wild type and had a reduced skeletal muscle mass (Kamei et al 2004). A recent study reported *FOXO1* as a strong candidate for daily gains and breast muscle weight in chicken (Xie et al. 2012). We also noticed a differentiation peak overlapping the *SMPD3* gene (max Fst = 0.95, *P* = 0.00001) on GGA11. *SMPD3* is shown to be genetically casual for developmental defects, including dwarfism and delayed puberty (Stoffel et al. 2005). Inactivation of *SMPD3* protein is also reported to be associated with skeletal deformities, fractures, and developmental defects of bone (Aubin et al. 2005).

#### Selection Candidates for Appearance Traits

Another candidate selective sweep was localized over the *SOX5* gene (max Fst = 0.55, *P* = 0.00429) on GGA1 that causes the Pea-comb phenotype in chickens (Wright et al. 2009). Pea-comb is a dominant mutation in chickens that drastically reduces the size of comb and wattles. It is an adaptive trait in cold climates as it reduces heat loss and makes the chicken less susceptible to frost lesions (Wright et al. 2009). Pea-combed Indian game or so-called Cornish roosters have conventionally been used as sire line in commercial broilers for the past 50 years. In comparison, Leghorns, the most dominant egg layers, have single or rose forms of comb that explain why Fst in the *SOX5* gene is elevated. Therefore, the *SOX5* along with the aforementioned *BCO2* for yellow skin color are two genes detected in association with appearance traits in modern chicken.

Table 2 provides a partial list of candidate genes revealed by analysis of parallel divergence. The complete list of 170 regions with empirical *P* value < 0.01 for each region is provided in supplementary table S1, Supplementary Material online. The observation of multiple signals in highly selected populations of chicken is consistent with the hypothesis that production traits have a complex nature controlled by many genes. It also indicates the significant role of adaptation in shaping the chicken genome due to the widespread diversity observed among populations of modern chicken such that the current catalog of positively selected loci identified represents only the tip of the selective iceberg. An in-depth understanding of these genomic regions, for instance by defining the selected phenotype and underlying mutations, may provide concrete evidences for explaining parallel adaptation to different selection regimes. This is, however, not an easy task and beyond the scope of this study as selected regions mostly span over tens or hundreds of kilobases with multiple missense mutations or involve sequences with unknown functions that implicate dissecting the potential role of each selection signal.

Still, other implications exist regarding data provision that potentially affects the results. For example, sequencing has been done with different techniques and with unequal number of individuals and sequencing depth in layers and broilers. Furthermore, broilers are sequenced as a pool in contrary to the layers that are sequenced individually. Pooled sequences suffer uncertainty and incompleteness of data profile (Cutler and Jensen 2010). This uncertainty is even more severe for low frequency or rare alleles, where often are excluded from the analysis by setting a subjective threshold. This way, rare alleles will be heavily underrepresented, including those likely affected by selective forces. Future research with individually sequenced broilers will allow examination of candidate regions based on haplotype properties. Such efforts are currently underway by the authors, along with sequencing a sizable battery of red jungle fowl, the progenitor of modern chicken.

## Conclusion

This is the first attempt for localizing footprints of recent selection in commercial chicken based on individual full resequencing data. We found genetic parallelism associated with the selective pressure during domestication and probably recent improvement of chicken. We highlighted signatures of possible selection at 12 genes/regions relevant to appearance (e.g., *SOX5* along *BCDO2*) and production traits, exemplified by a striking evidence of selection at *OPG*, a gene involved in Osteoporosis a disorder related to overconsumption of calcium in egg-laying hens.

## Supplementary Material

Supplementary table S1 is available at *Genome Biology and Evolution* online (http://www.gbe.oxfordjournals.org/).

## Ethics Statement

Samples were collected by veterinarians in the LOHMANN Company in the course of a routine health check for diagnostic reasons and a partition of these samples was used to extract DNA. The authors collected no samples themselves.

**Table 2:** A Partial List of Candidate Gene/Regions Detected as Signal of Parallel Divergence

Chromosome	Start Base Pair	End Base Pair	Fst	*P*^a^	Gene	Function or Association
1	66066955	66119201	0.55	0.00429	*SOX5*	Pea comb
2	122495794	123015968	0.50	0.00525	*WWP1*	Muscular dystrophy
2	135771154	136193827	0.74	0.00071	*TNFRSF11B*, *EXT1*, and *EXT1L*	Osteoporosis and bone disorders
5	48161309	49265282	0.91	0.00004	*DLK1*	Muscle hypertrophy
9	19104947	19552350	0.64	0.00132	*CLDNS11*	Regulation of bone mineral density
11	15942	476726	0.95	0.00001	*SMPD3*	Dwarfism and delayed puberty

^a^Percentile of empirical distribution.

## Supplementary Material

Supplementary Data
