# Supplementary material for: Parallel Selection Revealed by Population Sequencing in Chicken
Source: Genome Biol Evol. 2015 Nov 13;7(12):3299–306. doi: 10.1093/gbe/evv222 (PMC4700953; doi:10.1093/gbe/evv222)
Supplement: Supplementary Data [file supp_7_12_3299__index.html]

Parallel selection revealed by population sequencing in chicken — Parallel Selection Revealed by Population Sequencing in Chicken — Supplementary Data 

# Parallel Selection Revealed by Population Sequencing in Chicken

## Supplementary Data

files

- Supplementary Data - txt file
